# Diagnosis and empirical treatment of fever of unknown origin (FUO) in adult neutropenic patients: guidelines of the Infectious Diseases Working Party (AGIHO) of the German Society of Hematology and Medical Oncology (DGHO)

**DOI:** 10.1007/s00277-017-3098-3

**Published:** 2017-08-30

**Authors:** W. J. Heinz, D. Buchheidt, M. Christopeit, M. von Lilienfeld-Toal, O. A. Cornely, H. Einsele, M. Karthaus, H. Link, R. Mahlberg, S. Neumann, H. Ostermann, O. Penack, M. Ruhnke, M. Sandherr, X. Schiel, J. J. Vehreschild, F. Weissinger, G. Maschmeyer

**Affiliations:** 1 Department of Internal Medicine II, University of Würzburg Medical Center, Würzburg, Germany; 2 Department of Internal Medicine-Hematology and Oncology, Mannheim University Hospital, Mannheim, Germany; 3 Department of Stem Cell Transplantation, University Hospital UKE, Hamburg, Germany; 4 Department of Hematology and Oncology, University Hospital of Jena, Jena, Germany; 5 Department I for Internal Medicine, University Hospital of Cologne, Cologne, Germany; 6 German Centre for Infection Research, partner site Bonn-Cologne, Cologne, Germany; 7 Clinical Trials Centre Cologne, ZKS Köln, Cölogne, Germany; 8 Center for Integrated Oncology CIO Köln-Bonn, Cologne, Germany; 9 Cologne Excellence Cluster on Cellular Stress Responses in Aging-Associated Diseases (CECAD), Medical Faculty, University of Cologne, Cologne, Germany; 10 Department of Hematology, Oncology and Palliative Care, Klinikum Neuperlach and Klinikum Harlaching, München, Germany; 11 Hematology and Medical Oncology Private Practice, Kaiserslautern, Germany; 12 Klinikum Mutterhaus der Borromäerinnen, Trier, Germany; 13 Medical Oncology, AMO MVZ, Wolfsburg, Germany; 14 Department of Hematology and Oncology, University of Munich, Munich, Germany; 15 Internal Medicine, Hematology, Oncology and Tumor Immunology, University Hospital Charité, Campus Virchow Klinikum, Berlin, Germany; 16 Department of Hematology and Oncology, Paracelsus-Klinik, Osnabrück, Germany; 17 Hematology and Oncology Practice, Weilheim, Germany; 18 Department of Hematology, Oncology and Palliative Care, Klinikum Harlaching, Munich, Germany; 19 Department of Internal Medicine, Hematology, Oncology and Palliative Care, Evangelisches Klinikum Bethel, Bielefeld, Germany; 20 Department of Hematology, Oncology and Palliative Care, Klinikum Ernst von Bergmann, Potsdam, Germany

**Keywords:** Neutropenia, Fever, Empirical therapy, Antibacterial, Antifungal, Infection

## Abstract

Fever may be the only clinical symptom at the onset of infection in neutropenic cancer patients undergoing myelosuppressive chemotherapy. A prompt and evidence-based diagnostic and therapeutic approach is mandatory. A systematic search of current literature was conducted, including only full papers and excluding allogeneic hematopoietic stem cell transplant recipients. Recommendations for diagnosis and therapy were developed by an expert panel and approved after plenary discussion by the AGIHO. Randomized clinical trials were mainly available for therapeutic decisions, and new diagnostic procedures have been introduced into clinical practice in the past decade. Stratification into a high-risk versus low-risk patient population is recommended. In high-risk patients, initial empirical antimicrobial therapy should be active against pathogens most commonly involved in microbiologically documented and most threatening infections, including *Pseudomonas aeruginosa*, but excluding coagulase-negative staphylococci. In patients whose expected duration of neutropenia is more than 7 days and who do not respond to first-line antibacterial treatment, specifically in the absence of mold-active antifungal prophylaxis, further therapy should be directed also against fungi, in particular *Aspergillus* species. With regard to antimicrobial stewardship, treatment duration after defervescence in persistently neutropenic patients must be critically reconsidered and the choice of anti-infective agents adjusted to local epidemiology. This guideline updates recommendations for diagnosis and empirical therapy of fever of unknown origin in adult neutropenic cancer patients in light of the challenges of antimicrobial stewardship.

**Electronic supplementary material:**

The online version of this article (doi:10.1007/s00277-017-3098-3) contains supplementary material, which is available to authorized users.

## Introduction

Neutropenic cancer patients have a high risk of infectious complications, depending on the extent and duration of neutropenia, as well as on additional cellular and/or humoral immunosuppression and disruption of skin and mucosal barriers. Duration and nadir of neutropenia are correlated with the incidence of fever and infections [1], which not only cause significant morbidity and mortality [2] but may also compromise further chemotherapy. At onset of fever of unknown origin (FUO) in neutropenic patients, prompt and effective evidence-based intervention is required.

In the past decade, an increasing rate of resistance among bacterial pathogens to widely used antibacterial agents, particularly beta-lactams and fluoroquinolones, has been noted. At the same time, only a small number of newer antimicrobial agents have become available, e.g., tigecycline, linezolid, moxifloxacin, cefozopran, telavancin, oritavancin, ceftazidime-avibactam, ceftolozan-tazobactam, or micafungin; however, data on their empirical use in neutropenic patients are limited or not existent. Efficacy and safety of anti-infective strategies have to be reassessed in this context. Newly introduced diagnostic tests and data on the usefulness of biomarkers for therapeutic decisions are to be critically reconsidered.

This guideline, an update of a version from 2003 [3], focuses on risk-adapted diagnostic procedures and empirical antimicrobial treatment in neutropenic cancer patients with FUO according to their likelihood of a complicated course of infection.

Separate AGIHO guidelines for hematological and oncological patients have been published on sepsis [4], primary prophylaxis of bacterial [5] and fungal infections [6], prevention of infections after allogeneic hematopoietic stem cell transplantation (HSCT) [7], diagnosis and treatment of invasive fungal infections [8, 9], management of pulmonary infiltrates [10], abdominal complications [11], venous catheter related infections [12], central nervous system infections [13], infections after autologous HSCT [14], and community respiratory viral infections [15].

### Guideline development, grading of recommendations, and definitions

A group of hematologists, oncologists, and infectious disease specialists was built within the AGIHO, which after thorough literature search (including only full publications but excluding allogeneic HSCT), created a set of core slides with statements and recommendations, discussed in face-to-face meetings, telephone conferences, and by electronic correspondence. The final version was approved in an AGIHO plenary meeting on 10 February 2017. This manuscript was reviewed by all co-authors. A detailed methodological report is provided in the Electronic supplementary material.

Consistent with recently updated AGIHO guidelines, the grading system currently used by the European Society for Clinical Microbiology and Infectious Diseases [16] (Table 1) was adapted.

**Table 1:** Grading system used in the present guideline (adapted from [16])

**Strength of recommendation**	**AGIHO**
Grade A	Strongly supports a recommendation for use
Grade B	Moderately supports a recommendation for use
Grade C	Marginally supports a recommendation for use
Grade D	Supports a recommendation against use
**Quality of evidence**	
Level I	Evidence from at least 1 properly designed randomized, controlled trial
Level II^a^	Evidence from at least 1 well-designed clinical trial, without randomization; from cohort or case-controlled analytic studies (preferably from ≥ 1 center); from multiple time series; or from dramatic results of uncontrolled experiences
Level III	Evidence from opinions of respected authorities, based on clinical experience, descriptive case studies, or reports of expert committees

^a^Added index: meta-analysis or systematic review of randomized controlled trials (*r*); transferred evidence, that is, results from different patient cohorts or similar immune-status situation (*t*); comparator group is a historical control (*h*), and uncontrolled trial (*u*)

## Definitions

### Neutropenia

There is no defined cut-off value for the neutrophil count clearly separating patients with or without increased risk of infections and mortality. In line with most recommendations and risk stratifications in clinical trials, a neutrophil count (segments and bands) < 500/μl or < 1000/μl with a predicted decline to < 500/μl within the next 2 days defines neutropenia.

### Fever

Different definitions of fever in neutropenia have been used in guidelines and clinical trials, and several methods and sites to determine the body temperature are available. In general, either a temperature measured orally of ≥ 38.3 °C once or ≥ 38.0 °C lasting for at least 1 h or being measured twice within 12 h or a method shown to be equivalent to these results may be used to define fever. In the absence of a definite non-infectious cause, such as a febrile reaction to cytokines, cytotoxic drugs (e.g., cytarabine or bleomycin), or a transfusion of blood products, this clinical symptom has to be regarded as a sign of an infectious complication. It should be kept in mind that fever may be obscured by antipyretic drugs used for analgesia or cancer treatment, such as prednisone, non-steroidal anti-inflammatory agents, or metamizole (dipyrone).

## Risk stratification

While it is widely accepted that the incidence of infections in cancer patients is directly related to nadir and duration of neutropenia [1], it is difficult to exactly predict this in an individual patient [17]. Clinical trials on treatment of fever and infections in patients with short periods of neutropenia, e.g., below 5 or 7 days, are limited, and some patients with neutropenia lasting for more than 5 days have also been enrolled in studies including oral therapy and outpatient care [18, 19]. As a result of a literature review, we have agreed upon stratification into two risk groups, i.e.,

- Standard risk: expected duration of neutropenia of up to 7 days and
- High risk: expected duration of neutropenia of at least 8 days.

However, while all patients with neutropenia lasting eight or more days are regarded as high-risk patients with respect to a complicated course of a febrile episode, those assigned to the standard-risk group may exhibit individual characteristics justifying their allocation to the high-risk population as well. These individual factors can be identified by the use of the Multinational Association of Supportive Care in Cancer (MASCC) criteria [17], which have been repeatedly validated [19–21] and are shown in Table 2.

**Table 2:** MASCC score to identify standard-risk patients with respect to a complicated course of a febrile episode [17]

Characteristic	Weight
Burden of febrile neutropenia with no or mild symptoms^a^	5
No hypotension (systolic blood pressure > 90 mmHg)	5
No chronic obstructive pulmonary disease	4
Solid tumor or hematologic malignancy with no previous fungal infection	4
No dehydration requiring parenteral fluids	3
Burden of febrile neutropenia with moderate symptoms^a^	3
Outpatient status	3
Age < 60 years	2

A score of ≥ 21 identifies a standard-risk patient

^a^Points attributed to the variable “burden of febrile neutropenia” are not cumulative and the maximum theoretical score is therefore 26

Standard-risk patients with a MASCC score of ≥ 21 constitute a group of patients with a high likelihood of a non-complicated clinical course of infection. Provided they meet all individual criteria listed in Table 3, primary outpatient management of neutropenic fever is possible (BIIr).

**Table 3:** Individual criteria to be fulfilled by patients to be treated primarily on an outpatient basis

General	No signs of CNS^a^ infection, severe pneumonia, or venous catheter infection
	No signs of sepsis or shock
	None of the following: associated organ failure, pronounced abdominal pain (±diarrhea), dehydration, recurrent vomiting, intravenous supportive therapy, necessity of permanent or close monitoring (e.g., metabolic decompensation, hypercalcemia)
	No new ECG abnormalities requiring treatment
	No new severe organ impairment
Oral antibiotics	No fluoroquinolone prophylaxis or therapy within the last 7 days
	Oral medication feasible
	Good compliance with oral medication expected
Outpatient management	Medical care ensured (different options)
	Patient does not live alone; patient/helpers have a telephone; patient can reach clinic skilled at treatment of neutropenic patients within 1 h
	Patient is conscious, knows, and understands the risks

^a^ *CNS*, central nervous system

## Epidemiology

### Most common pathogens identified in febrile neutropenic patients with microbiologically documented infections

At onset of fever, antibiotic therapy needs to be started immediately, and because of the time needed for microbiological tests, it will have to be empirical in the beginning in patients who also do not show a suspected clinical focus of infection. In about half of patients with febrile neutropenia, the antibiotic therapy will remain empirical, since no relevant pathogen or focus of infection can be identified during the following days [19, 22]. The grounds for selection of empirical antimicrobial agents are (a) reported results of prospective, randomized clinical studies and (b) microorganisms identified in patients with microbiologically documented infections by analogy. Here, *Staphylococcus aureus*, *Streptococcus* spp., enterococci, coagulase-negative staphylococci, gram-negative enterobacteria, and *Pseudomonas aeruginosa* are the most frequent and relevant pathogens [23, 24]. While numerically, coagulase-negative staphylococci are the most frequent microbial isolates in many institutions, a single blood culture positive for those commensal skin pathogens, in lack of a corresponding clinical focus of infection, should be considered contamination [25]. The same is true for other potential contaminants like *Corynebacterium*, *Bacillus cereus*, *Propionibacterium*, or *Micrococcus* spp*.* Among fungal pathogens, *Candida* spp. and *Aspergillus* spp. are predominant, the latter typically being associated with a prolonged duration of neutropenia in high-risk patients [26].

### Local epidemiology and impact of oral fluoroquinolone prophylaxis

A recent history of antibiotic prophylaxis or therapy increases the risk of infections due to bacterial pathogens resistant to the antibiotic used [27–29]. After ciprofloxacin prophylaxis, a relative predominance of infections caused by gram-positive cocci compared with gram-negative bacteria has been observed [30]. Quinolones have been reported as being associated with an increased rate of colonization by vancomycin-resistant enterococci (VRE) [31, 32] or methicillin-resistant *S. aureus* (MRSA) [33] and with a higher prevalence of multidrug resistance among enterobacteria via extended-spectrum beta-lactamases (ESBL) [34, 35]. Colonization by ESBL, VRE, or MRSA has been associated with an increased rate of bacteremia with these pathogens [32, 36–38]. As a consequence, the use of quinolones for interventional treatment in febrile neutropenic patients should be limited to microbiologically documented infections caused by in vitro susceptible microorganisms [39].

The local epidemiology must be taken into account for the appropriate choice of empirical antimicrobial therapy. Microbiological findings from patients treated in a defined hematology-oncology institution should be discussed on a regular basis, i.e., at least once a year, with infection-control and antimicrobial stewardship experts (BIII). Baseline screening of newly or re-admitted patients for multidrug-resistant pathogens, i.e., MRSA (BIII), VRE (BIII), and ESBL (BIIt), should be considered.

## Diagnosis

### Baseline diagnostic procedures before immunosuppressive therapy

Before starting myelosuppressive therapy, patients must be thoroughly explored for relevant previous or prevalent infections, which may become relevant during treatment-induced neutropenia (AIII). Clinical examination should be performed with special attention paid to skin, mucosa, puncture, and vascular catheter exit sites, paranasal sinuses, lungs, and the perianal region (AIII). In patients with a self-reported penicillin allergy, skin testing is recommended (BIIt), as a negative result (which is to be expected in the vast majority of cases) helps to avoid unnecessary first-line use of carbapenems, aztreonam, or vancomycin [40–42].

Baseline laboratory tests include a blood count, liver enzymes (ASAT/SGPT, ALAT/SGOT, gGT), total bilirubin, alkaline phosphatase, LDH, creatinine, blood urea nitrogen, coagulation tests (INR, aPTT), C-reactive protein, and urinalysis (BIII). Except for urinalysis, it is recommended to repeat these tests regularly, e.g., twice a week, during long-lasting neutropenia (BIII). Procalcitonin or cytokine levels (such as interleukin-6) are not recommended for routine baseline diagnostics (DIII).

If neither computed nor magnetic resonance tomography of chest and abdomen have been performed for staging of the underlying disease, chest radiographs (two views) and abdominal ultrasound may be considered a baseline examination before first chemotherapy to check for pre-existing abnormalities and facilitating comparison with subsequent studies (CIII). Particularly in high-risk patients, a thoracic CT scan prior to chemotherapy appears desirable for documentation of baseline status. However, in the absence of prospective studies, no recommendation can be made. In patients with a history of an invasive infection, appropriate imaging is recommended even in the absence of clinical symptoms of recurrence (BIII).

### Screening of asymptomatic neutropenic patients for invasive fungal infections

For high-risk patients with an expected duration of profound neutropenia > 7 days, serial (at least twice weekly) monitoring for *Aspergillus* galactomannan in serum has been recommended [43, 44]. Monitoring patients with 1,3-beta-d-glucan in blood samples [45, 46] is being discussed as an alternative but rarely used due to higher costs. A sensitive, validated *Aspergillus* PCR may also be helpful (CIII) for screening of blood samples in specific high-risk populations [47]. However, the sensitivity of these tests is strongly reduced in patients given systemic mold-active antifungals [48, 49], and false-positive results may be caused by beta-lactam antibiotics, parenteral nutrition, severe intestinal mucositis, or transfusion of blood products. Therefore, screening of afebrile and asymptomatic patients should be restricted to those not receiving systemic mold-active prophylaxis (BIIu). These non-culture-based procedures do not replace clinical, imaging, endoscopic, or other microbiological diagnostics (BIII). Details on early diagnosis of fungal infections are discussed in a separate guideline [8].

### Diagnostic procedures at onset of fever in neutropenia

Diagnostic measures at first fever in neutropenic patients aim at

- Ruling out non-infectious causes of fever
- Identifying a clinical focus and/or causative pathogens, and
- Assessing the severity of inflammatory response in order to early identify patients in need for intensive care

They must not delay the start of appropriate antibiotic therapy (AIIt) [50–53]. In a clinically unstable patient, eventually presenting in the emergency room, prompt start of antimicrobial therapy is required (AI) and immediate referral to an intensive care unit must be considered [8].

Thorough clinical examination must be updated (AIII) and repeated at least daily as long as a hospitalized patient is febrile (AIII). It may reveal a presumable focus of infection and enable a pre-emptive antimicrobial treatment targeting typically involved pathogens rather than purely empirical treatment (Table 4).

**Table 4:** Pathogens typically involved in clinically documented infections

Clinical signs and symptoms	Frequently involved pathogens
Erythema and/or pain at venous access	Coagulase-negative staphylococci
Mucosal ulcers	Alpha-hemolytic streptococci, *Candida* spp.
Single point-like skin lesions	Gram-positive cocci, *Candida* spp.
Necrotizing skin lesions	*Pseudomonas aeruginosa*, filamentous fungi
Diarrhea, meteorism	*Clostridium difficile*
Enterocolitis, perianal lesions	Polymicrobial (incl. anaerobes)
Lung infiltrates ± sinusitis	Filamentous fungi, *Pneumocystis jirovecii*
Retinal infiltrates	Candidemia

A minimum of two separate pairs of blood cultures must be taken prior to initiation of antibiotic therapy (AIII). There is no need to wait between sampling of cultures; separate sets can be achieved by venipuncture of both arms. If the patient has an indwelling central venous catheter (CVC), one pair should be drawn from a peripheral vein and at least one from the CVC. The diagnostic yield of this approach can be increased by taking a blood sample from each lumen of a CVC and by taking three pairs of blood cultures (60 ml blood) (BIIt) [54–56]. A “differential time to positivity” of ≥ 2 h between CVC and peripheral blood cultures may indicate a CVC-related infection [57, 58] and give reason for pre-emptive treatment described in a separate guideline [59] (BIIu). Multiplex PCR-based methods do not replace the standard microbiology (CIIu) [60–64] but may improve turnaround time, sensitivity, and specificity of pathogen detection [59]. A reduction in morbidity or mortality in febrile neutropenic patients through the use of PCR-based methods supplementing blood cultures has not yet been shown.

In addition to repeat baseline laboratory tests described above, determination of lactate, blood gas analysis, and coagulation tests, in order to early identify severe sepsis, should be considered (BIII). Biomarkers such as procalcitonin or interleukin-6 are widely used for assessing the severity of inflammation, but data on their prognostic or predictive value in adult patients with febrile neutropenia are conflicting [65–70]. In high-risk patients who did receive systemic mold-active antifungal prophylaxis and were not screened for *Aspergillus* galactomannan, beta-d-glucan, or fungal PCR, such a test (preferably galactomannan) should be ordered at this time to enable early detection of a breakthrough invasive fungal disease (BIII).

At onset of fever, a CT scan of the lungs is recommended in the case of respiratory tract symptoms (BIII). Conventional chest radiographs are discouraged (DIIt), as they show abnormalities in less than 2% of febrile neutropenic patients who have no clinical signs of lower respiratory tract infection [71–73]. Nasal congestion or signs and symptoms of sinusitis should give reason for a CT scan of paranasal sinuses (BIII) [74]. First data on PET-CT indicate a potential use for early identification of the source of fever/infection, particularly abdominal foci [75–78]. Despite these positive reports, an explicit recommendation for its routine use cannot be given due to the lack of systematic studies.

Gastrointestinal complaints or laboratory abnormalities should prompt abdominal ultrasonography (BIIu). An abdominal CT scan is an alternative if neutropenic enterocolitis is suspected (BIIu) [11, 79, 80].

## Antimicrobial therapy

The following recommendations are based on evidence from controlled studies and clinical experience. The status of approval by regulatory agencies and reimbursement policies have not been taken into account.

**Fig. 1:**
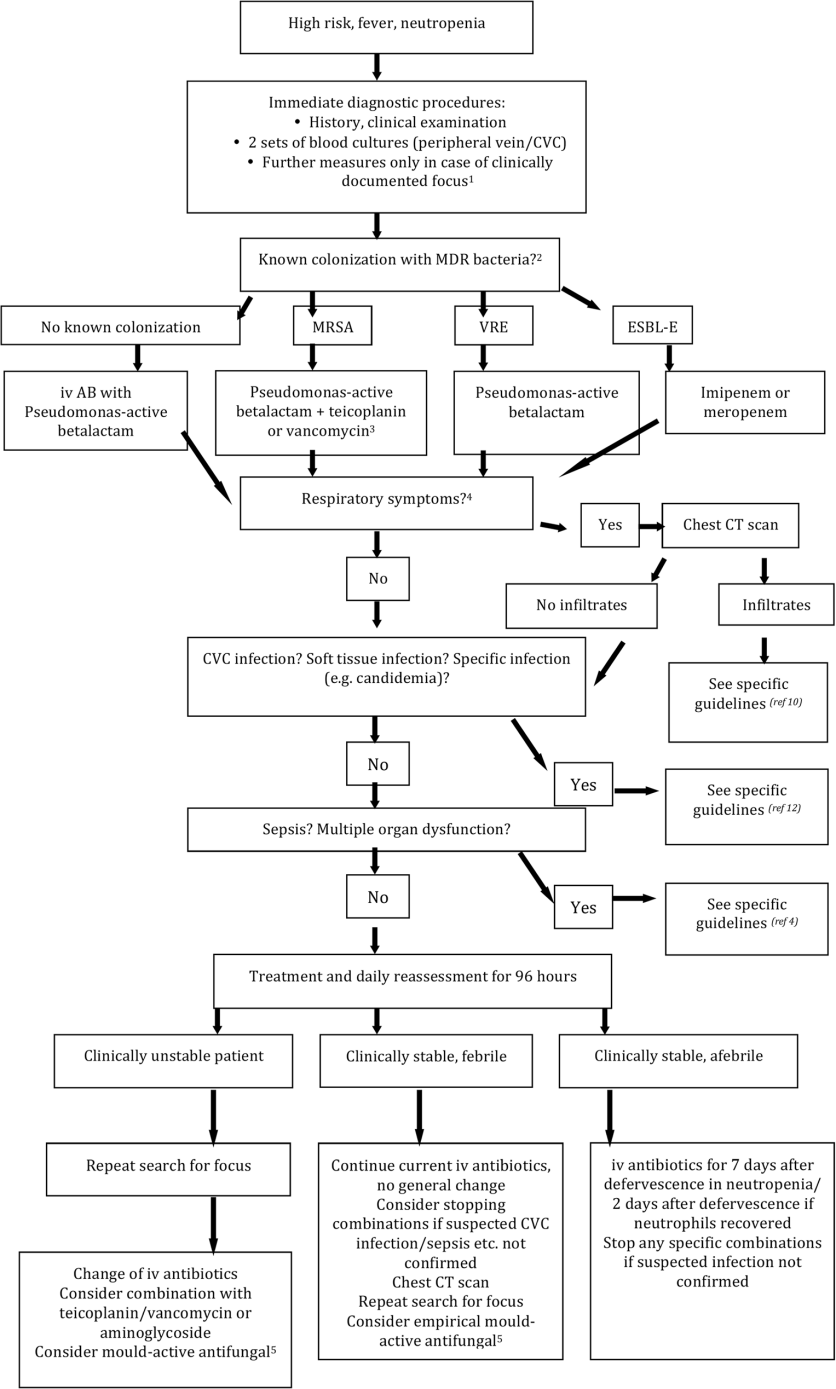
Treatment algorithm for febrile neutropenic high-risk patients: *1*, e.g., urine cultures, CT of sinuses, echocardiography, and viral PCR; *2*, in the case of carbapenem-resistant MDR bacteria individual choice according to in vitro susceptibility; *3*, monitor blood levels; *4*, e.g., tachypnea, dyspnea, cough, and pleuritic symptoms; *5*, strong recommendation for patients with high-risk neutropenia without mold-active prophylaxis. *CVC* central venous catheter, *MDR* multidrug resistant, *CT* computed tomography scan, *iv* intravenous, *AB* antibiotics, *MRSA* methicillin-resistant *Staphylococcus aureus*, *ESBL-E* extended spectrum beta-lactamase-producing enterobacteria, *VRE* vancomycin-resistant enterococci, *PCR* polymerase chain reaction

A clinical treatment algorithm for high-risk patients is depicted in Fig. 1.

### Antibacterial agents for empirical first-line therapy

In high-risk patients, the spectrum of first-line antibacterial agents should include gram-negative enterobacteria, *P. aeruginosa, S. aureus*, and streptococci (AI), while local epidemiology must be taken into account. Treatment must be started within 2 h after onset of fever, without awaiting the results of microbiology (AIIt) [50–53]. If oral fluoroquinolone prophylaxis has been given, it should be discontinued at the start of interventional antimicrobial therapy (AIII). Emergency treatment algorithms for this clinical situation as well as supervision or audits have been shown to improve timely and adequate therapy [81]. Piperacillin/tazobactam, imipenem, meropenem, cefepime,^1^ and ceftazidime are suitable for first-line empirical antibacterial monotherapy in severely neutropenic high-risk patients with FUO (AI). Most patients with a history of penicillin allergy will tolerate imipenem, meropenem, or an antipseudomonal cephalosporin. For the small cohort of patients with severe immediate-type hypersensitivity reaction, aztreonam [85] may be used as a—less well-studied—alternative (CIIu). In this setting, the addition of a vancomycin or teicoplanin to aztreonam may be considered due to the lack of activity of aztreonam against gram-positive bacteria (CIII) [86].For newer broad-spectrum antibacterial agents, such as ertapenem [87], which has insufficient activity against *Pseudomonas* spp., doripenem [88], ceftazidime-avibactam [89], ceftolozane-tazobactam [64, 90], or cefozopran [91], there is very limited data on their safety and efficacy for empirical treatment in adult febrile neutropenic cancer patients available so far. Tigecycline in combination with an antipseudomonal beta-lactam has shown benefit in terms of a lesser need for treatment modification in institutions with excess rates of multidrug-resistant pathogens [92] and for 2nd- or 3rd-line treatments [93].

### Antibacterial combination therapy

In high-risk patients, there is no evidence for superior efficacy of a combination of antibacterial agents compared with monotherapy (AIIr) [94]. Combining antibacterial agents in this indication has also not been shown to prevent the development of resistance [95]. A combination might be useful in institutions with a high prevalence of multidrug-resistant bacteria (AIIr) [92]. An antipseudomonal beta-lactam should always be included, with an aminoglycoside or a fluoroquinolone such as levofloxacin and ciprofloxacin as the combination partner (AIIt). For standard-risk patients without critically impaired renal function, the combination of an aminoglycoside with a third- or fourth-generation cephalosporin can be considered (AI) [96–98]. When aminoglycoside antibiotics are given, therapeutic drug monitoring is mandatory (AIIu) and once-daily dosing is appropriate (AIIr) [99].

A combination therapy including vancomycin or teicoplanin (DIIr) or linezolid (DIII) is generally discouraged for empirical first-line therapy [100] but might be considered in the case of (CIII) severe mucositis, skin or soft tissue infection, foreign body infection, or documented colonization of a patient with MRSA. The use of vancomycin is associated with an increased risk of nephrotoxicity, which should be diminished by therapeutic drug monitoring (BIIt). Although a higher rate of VRE infections has been found in VRE-colonized patients [31, 32, 101], the addition of linezolid to empirical first-line treatment has not shown a significant benefit [102]. Beyond this, the risk of thrombocytopenia as one of the major potential side effects of linezolid has to be considered.

### Empirical first-line antibacterial therapy in standard-risk patients with FUO

The recommendations in this paragraph affect patients with an expected duration of neutropenia ≤ 7 days who typically are not receiving systemic fluoroquinolone prophylaxis.

For patients with ≤ 7 days of neutropenia and no high-risk criteria identified by the MASCC score (Table 2) and without practical obstacles to outpatient management (Table 3), oral and outpatient therapy is recommended (AI) [103]. Hospitalization and in-patient start of antibacterial therapy, with a change to an outpatient treatment after defervescence or stabilization within the first 2 days, is an alternative approach validated in clinical studies (AI) [18, 19, 104].

Safety and feasibility of oral outpatient therapy for standard-risk patients identified by the MASCC score has recently been confirmed in a randomized prospective multicenter study, with less than 10% complications in the total study population and only 5% of patients requiring readmission under moxifloxacin monotherapy compared with a twice-daily combination of amoxicillin/clavulanate and ciprofloxacin [19]. For oral antibiotic therapy in standard-risk patients, a combination of amoxicillin/clavulanate with ciprofloxacin or monotherapy with moxifloxacin appears feasible (AI). Moxifloxacin is not active against *P. aeruginosa* [105, 106]. In the case of confirmed penicillin allergy, amoxicillin/clavulanate may be replaced by clindamycin [107] or cefuroxime axetil (BIIu) [108]. Primary intravenous therapy for patients of the standard-risk group may consist either of a monotherapy with ceftazidime, cefepime, or piperacillin/tazobactam, or, in the case of an increased rate of multiresistant gram-negative bacteria, of a combination of a third- or fourth-generation cephalosporin with an aminoglycoside (AI).

### Monitoring of inflammatory laboratory parameters during first-line empirical antibacterial therapy

Increasing CRP on day 5, compared with day 1, might indicate an unfavorable course of the episode [109]. Elevated procalcitonin may point at severe infection or sepsis in high-risk patients [65, 110–112]. Also, monitoring of procalcitonin on day 2 after onset of fever may help to detect a minority of patients with potentially severe infections, and in the case of persistent fever, it may contribute to early diagnosis of invasive mycoses [113]. Rising interleukin-6 typically indicates an unresolved infection/inflammation, while low levels have a high negative predictive value making severe septic infection unlikely [65–67, 114–117]. None of these parameters should be used alone for clinical decision-making (BIII).

### Re-evaluation of patients after ≥ 96 h of first-line empirical antibacterial therapy

After ≥ 96 h of persistent or recurrent fever despite adequate therapy, a multislice pulmonary CT scan (AIIu) [10, 118, 119] should be performed (preferably within 24 h after indication), independent of respiratory symptoms (AIIu). Other imaging procedures are indicated according to clinical signs or symptoms of a localized infection (BIIu). A thorough physical examination must be reiterated, with inspection of the oropharynx, skin lesions with particular attention to venous access and puncture sites and the perianal region, as well as painfulness of paranasal sinuses or other signs of upper airway infection (Table 4). Blood cultures from peripheral vein and indwelling central venous catheters should be repeated, while other microbiological cultures are only useful if clinical signs or symptoms indicate a possible site of infection (BIIu).

### Modifying antibiotic treatment in non-responders

If diagnostic procedures reveal a clinically documented infection or if a causative pathogen has been isolated, the empirical antibacterial approach should be changed to targeted or pre-emptive therapy (AIIt). Pre-emptive antimicrobial treatment is chosen according to the spectrum of microorganisms typically involved in the respective clinically documented infection (Table 4).

A change of the empirical antimicrobial treatment regimen can be considered in patients with fever recurrent or persisting for more than 96 h; however, a general change of antibacterial agents is not recommended (DIIr). In clinical studies on antibiotic therapy of neutropenic fever, median time to defervescence was 4 to 5 days [120–122]. The empirical addition of vancomycin after non-response to piperacillin/tazobactam [121] or teicoplanin after non-response to imipenem [123] has not been more effective than placebo, and defervescence after another 72 h of the unmodified beta-lactam regimen (placebo arms) was 45%. A modification or escalation of antimicrobial therapy only because of persistent elevation of inflammatory laboratory parameters has not been successful as well [70]. A change of antimicrobial therapy is recommended in patients with recurrent or persisting fever and clinical deterioration, instability, or other signs of progressive infectious disease (AIIu). In the case of severe sepsis and/or signs of critical organ failure, modification of antimicrobial therapy along with intensive further medical support is required (AIIu) [4].

As prospective studies for second-line antimicrobial therapy in neutropenic patients with persistent FUO under clearly specified 1st-line treatment regimens are sparse [124], recommendation of treatment modification are partially based on clinical expertise.

A change of empirical antimicrobial therapy aims to cover a broader range of bacteria and/or to overcome resistance among pathogens principally included in the spectrum of the first-line regimen. Again, the local prevalence of vancomycin-resistant enterococci, methicillin-resistant *S. aureus*, and extended-spectrum beta-lactamase-producing gram-negative bacilli, as well as the rate of primary resistance to piperacillin/tazobactam among *Escherichia coli* must be reconsidered (AIIt). Antimicrobial agents recommended for empirical second-line treatment are included in Table 5. In standard-risk patients initially treated with a cephalosporin plus/minus an aminoglycoside, a change to piperacillin/tazobactam, meropenem, or imipenem is recommended (AIIt).

**Table 5:** Antimicrobial agents suitable for 1st- and 2nd-line therapies

Risk groups		
	Standard risk (≤ 7 days)	High risk (≥ 8 days)
First-line	Outpatient therapy possible: • Amoxicillin/clavulanate + ciprofloxacin • Clindamycin + ciprofloxacin • Cefuroxime axetil + ciprofloxacin • Moxifloxacin	• Piperacillin/tazobactam • Ceftazidime, cefepime • Imipenem, meropenem
	Hospitalization required: • Ceftazidime, cefepime • Piperacillin/tazobactam • 3rd/4th-generation cephalosporin + aminoglycoside	
2nd-line, if indicated	• Imipenem, meropenem	• After piperacillin/tazobactam or ceftazidime or cefepime: imipenem, meropenem
	• After failure of outpatient regimen also consider piperacillin/tazobactam	• After imipenem or meropenem: addition of vancomycin or teicoplanin or aminoglycoside^a^ *plus* • Mold-active antifungal

^a^Depending on local epidemiology and individual patient-related risk factors

### Empirical antifungal treatment in high-risk patients

Empirical antifungal therapy is not recommended in patients of the standard-risk group (DIII). In high-risk patients, a prospective randomized trial showed a higher defervescence rate after addition of empirical mold-active antifungal therapy as compared with modification of antibacterial agents only [125]. These data were confirmed in a meta-analysis, yet a significant survival benefit could not be demonstrated [126, 127]. No benefit, however, could be shown for high-risk neutropenic patients, if antifungal therapy was applied already at onset of first fever instead of fever persisting for more than 72 h [128].

For high-risk patients without prior systemic antifungal prophylaxis, mold-active empirical antifungal therapy is recommended, if fever persists for ≥ 96 h or if fever relapses despite adequate antibacterial therapy (AI). This also includes patients given either oral itraconazole prophylaxis but not achieving sufficient serum or plasma trough concentrations (> 500 ng/ml) or a mold-inactive prophylaxis, i.e., fluconazole (BIIt). For patients receiving oral voriconazole or posaconazole prophylaxis, no prospective trial on the efficacy of a switch to another mold-active agent for empirical antifungal therapy is available. Thus, such a switch may be judicious in the setting of persistent FUO (CIII), but if a patient shows no clinical sign of invasive fungal disease despite adequate diagnostic work-up, blood samples were negative for *Aspergillus* galactomannan, and levels of posaconazole or voriconazole are within the target range, unmodified continuation of oral antifungal prophylaxis is reasonable. In the case of clinical deterioration, a change to an intravenously applied antifungal agent is recommended (AIII). For empirical mold-active antifungal therapy in febrile neutropenic patients, caspofungin and liposomal amphotericin B (AmB) are approved [129, 130]. Liposomal amphotericin B is preferred in patients at increased risk of fungal infection with non-*Aspergillus* molds (AI).

Several studies have aimed at a reduction of antifungal therapy in high-risk patients by not empirically treating all patients with persisting fever in prolonged neutropenia but only those with additional findings indicating the presence of a fungal disease. Utilizing pulmonary CT scan and testing for galactomannan, *Aspergillus*-specific PCR or both have been used for this so-called diagnostic-driven or pre-emptive approach. An increased number of invasive fungal infections and a substantially reduced consumption of antifungals were found in the pre-emptive as compared with the empirical treatment groups, without a significant increase in mortality rates [127, 131–135]. This approach cannot be recommended as a routine standard but might provide an alternative to empirical antifungal therapy (BIIr).

Numerous studies have compared efficacy and safety of empirical antifungal treatment. The most robust data are available for caspofungin or liposomal AmB (AI) (Table 6). Conventional AmB deoxycholate is not recommended because of its renal toxicity and other adverse events (DI). The use of the two lipid AmB formulations ABCD and ABLC is not supported due to the lack of appropriate clinical studies in this setting. For voriconazole (BI), a prospective trial could not demonstrate non-inferiority to liposomal AmB in a composite endpoint, although it was associated with a lower rate of breakthrough fungal infections [136]. For itraconazole, relevant data are available for the intravenous formulation [137]. As the oral application is associated with impaired bioavailability, only the intravenous application can be recommended (BI). For micafungin (CI**)**, a comparison with intravenous itraconazole showed superior response rates, but no study with a standard of care (caspofungin or liposomal AmB) as the comparator is available [138–141]. No data are available for anidulafungin or posaconazole in the empirical indication. Fluconazole, with no effect on mold infections, is not recommended for empirical antifungal therapy in persistently febrile neutropenic high-risk patients [125].

**Table 6:** Recommendations for empirical antifungal therapy in high-risk neutropenic patients without prior *Aspergillus*-active antifungal prophylaxis and fever persisting for ≥ 96 h

	Level	Evidence
cAmB	D	I
ABLC	D	I
ABCD	D	I
L-AmB	A	I
Caspofungin	A	I
Itraconazole IV	C	I
Micafungin	C	I
Voriconazole	B	I

*c-AmB* conventional amphotericin B (= deoxycholate AmB), *ABCD* amphotericin B colloidal dispersion, *ABLC* amphotericin B lipid complex, *L-AmB* liposomal amphotericin B, *IV* intravenous

### Empirical antiviral treatment

Empirical antiviral therapy in febrile neutropenic patients without signs or symptoms typical for a viral infection is discouraged (DIII).

## Adjunctive measures

### Granulocyte colony-stimulating factor

The adjunctive use if granulocyte colony-stimulating factor (G-CSF) is not recommended for routine clinical practice in febrile neutropenic patients (DIIr). If G-CSF has not been started before the onset of neutropenia, its interventional use can be considered in patients with fever and neutropenia who are at high risk for infection-associated complications or who have prognostic factors that are predictive of poor clinical outcomes, including expected prolonged (**>** 10 days) and profound (**<** 100/μl) neutropenia, age **>** 65 years, uncontrolled primary disease, or hospitalization at the time of fever development [142] (BIIr).

### Polyclonal immunoglobulins

Supportive therapy with polyclonal immunoglobulin is recommended only in select neutropenic patients with proven immunoglobulin deficiency (BIIt).

### Removal or change of a central venous catheter and hygiene

In a high-risk setting such as neutropenic fever, potential sources of infection should be identified and removed if possible. Central venous catheters not indispensable for patient care should be taken off. In patients with persistent FUO in whom no focus of infection has been found, empirical removal or change of the catheter may be justified (CIII).

## Duration of empirical antimicrobial therapy after defervescence

The appropriate duration of antimicrobial therapy in neutropenic patients after onset of stable defervescence, i.e., body temperature below 38 °C without the use of antipyretic drugs, has not been prospectively studied. A continuation until neutrophil recovery [143] has been questioned by results from several studies [22, 125, 144, 145] on discontinuation of parenteral antibiotics in responding, but persistently neutropenic patients, showing no substantial rates of recurrence of fever or documented infections. Challenges of antimicrobial stewardship in an era of globally increasing multidrug-resistance and missing development of new broad-spectrum anti-infectives gave reason for the recommendation to drastically shorten the administration of antibiotics in neutropenic patients after treatment response [146].

In persistently neutropenic patients, empirical therapy may be discontinued, but not earlier than 7 days after the onset of stable defervescence (without the use of antipyretic agents) and only in the absence of clinical signs or symptoms of infection (BIII). In this setting, a re-institution of systemic antibacterial prophylaxis, if given before the onset of fever, may be considered (CIII) [147]. In the case of hematopoietic recovery to a neutrophil count of >500/μl, empirical antimicrobial therapy can be safely discontinued after 2 days of stable defervescence [22] (BIII).

## Summary of recommendations Tables 7, 8, 9, and 10

**Table 7:** Summary of recommendations for diagnostic procedures in asymptomatic high-risk patients before onset of neutropenia

Patient population	Intention	Intervention	Strength of recommendation	Quality of evidence
High-risk neutropenia	Identify previous infection	Take history and perform physical examination	A	III
High-risk neutropenia	Identify previous infection	Order chest radiograph (2 views)^a^	C	III
High-risk neutropenia	Identify previous infection	Order abdominal ultrasound^a^	C	III
High-risk neutropenia	Identify colonization with VRE or MRSA	Take nasal/pharyngeal (MRSA) or rectal (VRE) swabs	B	III
High-risk neutropenia	Identify colonization with ESBL	Take rectal swabs	B	IIt

^a^In patients without recent chest/abdominal CT scan performed to stage the underlying disease

**Table 8:** Summary of recommendations for diagnostic procedures in neutropenic patients with fever

Patient population	Intention	Intervention	Strength of recommendation	Quality of evidence
Febrile neutropenia	Identify focus of infection	Take history and perform physical examination	A	III
Febrile neutropenia	Diagnose blood stream infection	Take at least 2 separate sets of blood cultures (BC) prior to start of antimicrobial therapy	A	II
Febrile neutropenia, indwelling central venous catheter (CVC)	Diagnose CVC infection	Take at least 1 set of BC from peripheral vein and 1 set of BC from CVC	A	II
Febrile neutropenia, no respiratory symptoms	Diagnose pneumonia	Order chest radiograph	D	II
Febrile neutropenia, respiratory symptoms	Diagnose pneumonia	Order thoracic CT scan	B	III
Persistent (≥ 96 h) febrile neutropenia	Diagnose pneumonia	Order thoracic CT scan	B	II

**Table 9:** Summary of recommendations for antimicrobial treatment of FUO

Patient population	Intention	Intervention	Strength of recommendation	Quality of evidence
Febrile neutropenia	Cure	Start antibiotic therapy (ABT) within 2 h	A	III
Outpatient febrile neutropenia, standard risk	Cure	Consider oral ABT with amoxicillin/clavulanate + ciprofloxacin or with moxifloxacin	A	I
High-risk febrile neutropenia	Cure	Intravenous ABT with piperacillin/tazobactam, imipenem, meropenem, cefepime, or ceftazidime	A	I
Persistent (≥ 96 h) high-risk febrile neutropenia, no mold-active prophylaxis	Cure	Empirical antifungal therapy with caspofungin or liposomal amphotericin B	A	I

**Table 10:** Daily dosages of antimicrobial agents in adult febrile neutropenic patients without specific contraindications or renal dysfunction

Substance	Application	Dosage
Amoxicillin/clavulanate	Oral	1000 mg twice or 3 times daily
Ciprofloxacin	Oral	500–750 mg twice daily
Levofloxacin	Oral	500 mg twice daily
Moxifloxacin	Oral	400 mg once daily
Piperacillin/tazobactam	Intravenous	4.5 g 3 or 4 times daily
Meropenem	Intravenous	1 g 3 times daily
Imipenem	Intravenous	0.5–1 g 4 times daily
Ceftazidime	Intravenous	2 g 3 times daily
Cefepime	Intravenous	2 g 3 times daily
Gentamicin^a^	Intravenous	1.5–2.0 mg/kg 3 times daily or 4.5–6.0 mg/kg once daily
Tobramycin^a^	Intravenous	1.5–2.0 mg/kg 3 times daily or 5.0–6.0 mg/kg once daily
Amikacin^a^	Intravenous	7.5 mg/kg twice daily or 15 mg/kg once daily
Vancomycin^a^	Intravenous	1 g twice daily
Teicoplanin^b^	Intravenous	400 mg once daily with one additional loading dose 12 h after the first dose
Caspofungin	Intravenous	70 mg day 1, 50 mg once daily from day 2 onwards
Liposomal amphotericin B	Intravenous	3 mg/kg once daily

^a^Therapeutic drug monitoring required

^b^Therapeutic drug monitoring recommended in selected patients

## Electronic supplementary material

ESM 1 (DOCX 21 kb)

## Footnotes

1 A first meta-analysis of cefepime in this setting had indicated an excess mortality compared with other antibiotics [82]; however, after data re-evaluation by the US-FDA, this was not confirmed and approval was not changed [83, 84].
