# Supplementary material for: Diagnosis and empirical treatment of fever of unknown origin (FUO) in adult neutropenic patients: guidelines of the Infectious Diseases Working Party (AGIHO) of the German Society of Hematology and Medical Oncology (DGHO)
Source: Ann Hematol. 2017 Aug 30;96(11):1775–92. doi: 10.1007/s00277-017-3098-3 (PMC5645428; doi:10.1007/s00277-017-3098-3)
Supplement: Supplementary file 1 — (DOCX 21 kb) [file 277_2017_3098_MOESM1_ESM.docx]

# Guideline Report:

# Diagnosis and empirical treatment of fever of unknown origin (FUO) in adult neutropenic patients. Guidelines of the Infectious Diseases Working Party (AGIHO) of the German Society of Hematology and Medical Oncology (DGHO)

# Reasons for Writing this Guideline

Especially in the fields of haematology/oncology and infectious diseases, standards of care can differ substantially between countries and regions. For example, there is substantial variation in the epidemiology of malignancies, multi-resistant bacteria, and opportunistic infections. The decision for inpatient or outpatient treatment depends upon available resources, population density, and social networks. Acceptance of death, desperate prognosis, and treatment discontinuation is dependent on many socio-cultural factors.

While existing international guidelines are a valuable reference, treatment recommendations for cancer and infections must factor in local circumstances. The German Society for Hematology and Medical Oncology (DGHO) aims at providing free up-to-date guidance to German hematologists and oncologists independent of treatment scenario or place of work. As part of the DGHO, the Infectious Diseases Working Party in Hematology and Oncology (AGIHO) specializes in guidelines on infection-related issues in hematological and oncological patients as well as hematopoietic stem cell transplant recipients. Numerous guidelines have been published as a result of this cooperation.

# Intended Audience

This guideline appeals to:

- Hematologists / oncologists and other physicians prescribing anti-cancer chemotherapy in hospital, outpatient services or private oncology practice (primary target group)
- Other physicians, nursing, and service staff caring for patients under treatment for malignant diseases
- Patients, policy makers, and insurance companies as a guidance for best practice

# Aim of this Guideline

This guideline will provide evidence-based recommendations on the use of diagnostic procedures, antimicrobial agents and adjunctive measures for empirical treatment in febrile neutropenic patients with malignancies. Specific therapeutic intervention in documented infections such as sepsis, venous catheter-associated infection, lung infiltrates or invasive fungal infection have been addressed in specific other AGIHO guidelines.

# Composition and Conduct of the Expert Panel, Consensus Procedure

Membership in the AGIHO is free for all DGHO members. DGHO membership status can be achieved by receiving two recommendations by existing members (peers). There are multiple annual meetings of the AGIHO; members are invited at least four weeks ahead. A full agenda of these meetings is part of the invitation, so all members are informed of guidelines brought up for discussion. The discussion is open for all members and every member present has one vote, with the limitation that only physicians in patient care are allowed to vote. Physicians employed by pharmaceutical companies are excluded from voting.

For this guideline, a group of haematologists/oncologists, hematopoietic stem cell transplant experts and infectious diseases experts was formed within the AGIHO. The co-ordinator was elected by this group in a written voting procedure. A Medline search for English and German language publications in the field of diagnosis and treatment of fever in neutropenic patients, from end of preparation for of the previous guideline in 2002 up to January 2013, was conducted by all co-authors and complemented by additional referenced and further literature. Only data from full publications (no meeting abstracts) were included. The search for publications on antimicrobial therapy included, but was not limited to, a systemic user-defined retrieval of the PubMed database, using combinations of search terms (empiric/empirical, antibiotic, infection, neutropenia/neutropenic, fever, antifungal, treatment/therapy). First, evidence tables according to available literature were created. The authors then drafted recommendations including evidence gradings and presented them to the panel for further intensive discussion and final decision on the grading and exact wording of the recommendation. If unanimity could not be achieved through discussion, decisions were made by majority vote of the panel. The consensus process was carried out via telephone conferences, an e-mail-based electronic discussion, and two face-to-face meetings, including a final consensus meeting and approval of recommendations by the guideline assembly of AGIHO on February 10^th^ 2017. Levels of evidence were ranked based on the classification of the European Society of Clinical Microbiology and Infectious Diseases (Table 1).

| **Strength of recommendation** | **AGIHO** |
| --- | --- |
| Grade A | strongly supports a recommendation for use |
| Grade B | moderately supports a recommendation for use |
| Grade C | marginally supports a recommendation for use |
| Grade D | supports a recommendation against use |
| **Quality of evidence** |  |
| Level I | Evidence from at least one properly designed randomized, controlled trial |
| Level II* | Evidence from at least one well-designed clinical trial, without randomization; from cohort or case-controlled analytic studies (preferably from ≥1 center); from multiple time series; or from dramatic results of uncontrolled experiences |
| Level III | Evidence from opinions of respected authorities, based on clinical experience, descriptive case studies, or reports of expert committees |

*Added index:

r: meta-analysis or systematic review of randomized controlled trials.

t: transferred evidence, that is, results from different patients’ cohorts, or similar immune-status situation.

h: comparator group is a historical control.

u: uncontrolled trial.

**Table 1**. Grading system used in the present guideline (adapted from [[16](#_ENREF_16)])
